# Effect of settling time and organic loading rates on aerobic granulation processes treating high strength wastewater

**DOI:** 10.1016/j.heliyon.2024.e36018

**Published:** 2024-08-09

**Authors:** Kyung Jin Min, Eunyoung Lee, Ah Hyun Lee, Do Yeon Kim, Ki Young Park

**Affiliations:** a Department of Tech Center for Research Facilities, Konkuk University, 120 Neungdong-ro, Gwangjin-gu, Seoul, 05029, South Korea; b Department of Civil and Environmental Engineering, Konkuk University, 120 Neungdong-ro, Gwangjin-gu, Seoul, 05029, South Korea

**Keywords:** Aerobic granule sludge, Organic loading rate, Settling time, Extracellular polymeric substance

## Abstract

Despite its numerous advantages, the aerobic granular sludge (AGS) process faces several challenges that hinder its widespread implementation. One such challenge is the requirement for high organic load ratios (OLR), which significantly impacts AGS formation and stability, posing a barrier to commercialization. In response to these challenges, this study investigates the granulation and treatment efficacy of the AGS process for treating high-concentration wastewater under various OLR and settling time. Three sequential batch reactors (R1, R2, R3) were operated at OLRs of 0.167, 0.33, and 1 kg COD/m^3^·day. The study focuses on analyzing key parameters including sludge characteristics, extracellular polymeric substances (EPS) content, PN/PS ratio, and microbial clusters. Results demonstrate that reducing settling time from 90 to 30 min enhances sludge settleability, resulting in a maximum 50.8 % decrease in SVI_30_ (from 98.1 to 122.8 mL/g to 51.9–81.3 mL/g), thereby facilitating the selection of beneficial microorganisms during granulation. Particularly, at R2, the PN/PS ratio was 4.3, and EPS content increased by 1.52-fold, leading to a 1.41-fold increase in sludge attachment. This observation suggests a progressive maturation of AGS. Additionally, analysis of microbial diversity and cluster composition highlights the influence of OLR variations on the ratios of *Proteobacteria* and *Bacteroidetes*. These findings emphasize the significant impact of SBR operational strategies on AGS process performance and biological stability, offering valuable insights for the efficient operation of future high-concentration wastewater treatment processes.

## 1 Introduction

Aerobic granular sludge (AGS) technology is recognized for its ability to self-agglomerate microorganisms into granular aggregates, making it a highly effective method for wastewater treatment. Its advantages include a compact structure, efficient settling performance, resilience to shocks, and high nitrogen removal efficiency [1]. Despite these merits, challenges persist in AGS technology, including prolonged granule formation, operational instability, and susceptibility to collapse. Current efforts to expedite AGS formation and enhance stability involve manipulating operating parameters, adding ions, and microbial inoculation. Among these approaches, controlling operating parameters is considered fundamental and critical compared to using exogenous substances [2].

The organic loading rate (OLR) significantly influences the formation and stability of AGS. A low OLR prolongs the granule formation period but results in small and stable particles. Conversely, a high OLR accelerates granule formation, but excessive microbial growth can lead to the formation of unstable granules [3]. In a study by Zhang et al. [4], AGS operated under high OLR conditions formed relatively larger and faster compared to conditions with low OLR. The size of activated sludge flocs generated in conventional activated sludge processes is proportional to the organic loading rate. A sudden increase in organic loading rate increases the size and density of AGS but reduces microbial species diversity, leading to a decrease in the solidity of the three-dimensional structure. Therefore, supplying appropriate organic loads is crucial for the vigorous formation and maintenance of heterotrophic granular sludge.

Recent studies on AGS have primarily focused on high or moderate OLRs [5,6]. However, municipal wastewater typically features low OLRs (0.28–2 kg COD/m^3^·day). Additionally, various types of real wastewater, such as side-stream from sewage treatment plants, fertilizer production wastewater, and leachate from landfills, generally have high nitrogen concentrations relative to COD. Consequently, operation under conditions of low OLR and high nitrogen loading rates is inevitable. Nevertheless, the operational feasibility of AGS processes under low OLR, such as 0.15 kg COD/m^3^·day, has been scarcely reported, particularly in nitrogen-rich wastewater. Moreover, intracellular protein hydrolysis, anaerobic granule core degradation, and the overgrowth of filamentous microorganisms are major causes of instability in AGS under high-concentration wastewater conditions [7]. Hamza et al. [8] found that culturing aerobic granular sludge at a high influent COD concentration of 4500 mg/L resulted in excessive biomass growth, negatively impacting the stability of AGS by causing the presence of methane-generating substances in the sludge core, leading to its degradation.

Among the parameters, the OLR plays a crucial role as an essential energy source for maintaining microbial activity and influences the formation and structural stability of AGS, while operational conditions such as settling velocity, shear force, and settling time can be key factors promoting granulation [9]. Settling time not only affects the formation of AGS but also plays a crucial role in regulating the long-term operational stability of granular sludge [10]. Peng et al. [11] reported that by employing strategies to reduce settling time, AGS reactors could be operated within 40 days with a sludge granulation degree of 90.22 %. While various studies are underway to promote AGS formation through diverse parameters such as inoculum sludge, substrate characteristics, organic loading, pH, temperature, and reactor operating conditions, cases of operating under low OLR conditions with high-concentration wastewater have not yet been reported.

This study aims to promote the formation of AGS for treating high-strength wastewater under two easily applicable field parameters: OLR and settling time. The main objective is to ensure stable operation and AGS formation by providing gradual changes. To achieve this goal, the study investigates sludge characteristics, changes in extracellular polymeric substances (EPS) content and composition, sludge adhesion, and microbial community changes in three sequential batch reactors (SBRs) operated under different OLR conditions. By identifying the effects of low OLR conditions on process performance and sludge characteristics, this study could provide a promising alternative for the treatment of high-strength wastewater containing high concentrations of organic matter and nitrogen.

## 2 Material and methods

### 2.1 Experimental design and operation

**Fig. 1:**
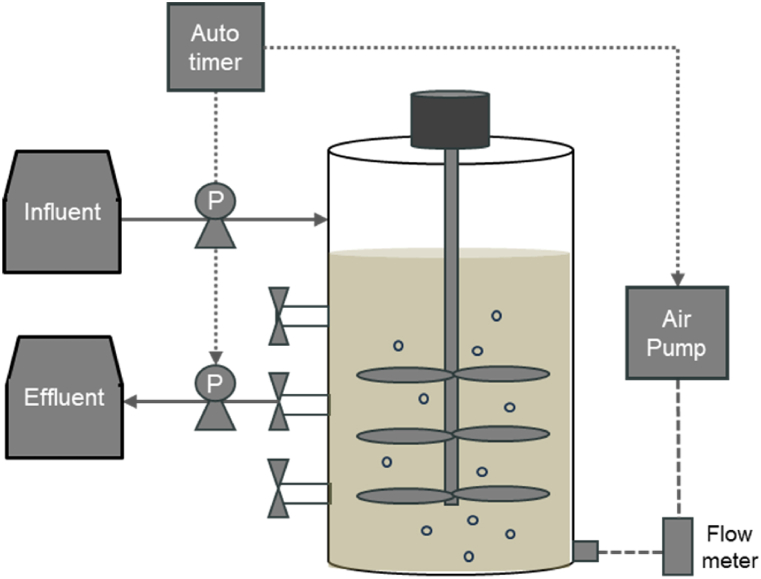
Schematic diagram of the lab-scale sequencing batch reactor setup.

In this study, three SBRs with an effective volume of 3 L each were utilized, operating under distinct OLR conditions. The OLR conditions for the three reactors were set at 0.167, 0.33, and 1 kg COD/m^3^·day, designated as R1, R2, and R3, respectively. Corresponding to these OLR conditions, the volume exchange rates were 3.3 %, 6.7 %, and 20 %, respectively. The operational temperature was maintained at 28 ± 2 °C, while the pH was regulated within the range of 7.0–8.5. In instances where the pH dropped below 7.0, sodium bicarbonate (NaHCO_3_) from Samchun Pure Chemical Co., Ltd., Korea, was added to the reactor to sustain a pH above 7.0. The schematic representation of the SBR reactors is depicted in Fig. 1. For aeration under aerobic conditions, air was introduced from the bottom of the reactor utilizing an air pump (LP-60A, Kosung Valve Co., Ltd., Korea), while a stainless-steel stirrer facilitated mixing. The operational sequences of the SBRs were regulated through an automatic timer, managing influent addition (mixing), aeration, settling, and idling phases.

The experiment was structured into two phases, delineated by the operating conditions observed throughout the entire operation period (Table 1). Phase I (0–37 days) entailed 24-h cycles comprising four influent (mixing) stages, four aeration stages, one settling stage, and one effluent discharge stage per cycle, with the following time allocations: mixing for 1 h (including 10 min of influent addition), aeration for 4.5 h, settling for 1.5 h, and idling for 0.5 h. Phase II (38–122 days) mirrored the cycle composition and duration of Phase I, albeit with a reduced settling time of 0.5 h. To offset this reduction, the mixing time was proportionally increased.

**Table 1:** SBR operating conditions.

Phase	Time (d)	Cycle (hr)			
		Mixing^a^	Aeration	Settling	Idling
Ⅰ	0–37	1	4.5	1.5	0.5
Ⅱ	38–122	1.25	4.5	0.5	0.5

a including 10 min of influent.

### 2.2 Inoculum and synthetic wastewater

The inoculum utilized in this study was sourced from the activated sludge of a biological reactor treating side-stream in a biogas plant situated in Yeongcheon City, South Korea, with MLSS 9290 mg/L, MLVSS 6640 mg/L and SVI_30_ 221 mL/g. Synthetic wastewater, designed to emulate side-stream rich in organic matter and nitrogen, served as the influent. The synthetic wastewater composition comprised sodium acetate (CH_3_COONa) from Samchun Pure Chemical Co., Ltd., Korea, at a concentration of 6.6 g/L as the carbon source (COD 5000 mg/L), NH_4_Cl as the nitrogen source, and KH_2_PO_4_ as the phosphorus source, with NH_4_Cl at a concentration of 11.46 g/L (NH_4_^+^-N 3000 mg/L), KH_2_PO_4_ at 131.8 mg/L (PO_4_^3-^-P 30 mg/L), CaCl_2_ · 2H_2_O 30 mg/L, MgSO_4_ · 7H_2_O 25 mg/L, FeSO_4_ · 7H_2_O 20 mg/L, and trace elements at 1 mL/L (H_3_BO_3_ 0.05 g/L, ZnCl_2_ 0.05 g/L, CuCl_2_ 0.03 g/L, MnSO4 · H_2_O 0.05 g/L, (NH_4_)_6_Mo_7_O_24_ · 4H_2_O 0.05 g/L, AlCl_3_ 0.05 g/L, CoCl_2_ · 6H_2_O 0.05 g/L, NiCl_2_ 0.05 g/L).

### 2.3 Analytical methods

Throughout the operational period, the reactors' COD, T-N, NH_4_^+^-N, NO_2_^−^-N, NO_3_^−^-N, mixed liquor volatile suspended solids (MLVSS), and SVI_30_ were analyzed following Standard Methods [12]. Periodic particle size analysis of the sludge was performed using a particle size analyzer (Malvern Mastersizer 2000, Malvern Panalytical Ltd., Malvern, UK), while EPS extraction from the sludge was conducted using the thermal extraction method outlined in previous studies [13]. The composition of the extracted EPS was assessed using the Bradford method for polysaccharides and the Lowry method in conjunction with the Bio-Rad DC protein assay for proteins. To characterize the organic matter composition, EPS samples underwent analysis using Fluorescence excitation–emission matrix (F-EEM) with an RF-5301 spectrofluorometer (Shimadzu Co., Japan). The fluorescence characteristics of organic matter were scanned utilizing an arc lamp with excitation wavelengths ranging from 220 to 400 nm (in 10 nm intervals) and emission wavelengths ranging from 280 to 600 nm (in 1 nm intervals).

For the analysis and quantification of biofilm formation and characteristics, sludge adhesion experiments were conducted as described by Song et al. [14]. Polyvinylidene fluoride (PVDF) membrane pieces measuring 150 mm × 150 mm were prepared and incubated in the sludge suspension with agitation at 150 rpm for 24 h. The microbial-attached membrane pieces were subsequently rinsed twice with 0.9 % NaCl solution and stained using the LIVE/DEAD BacLight Bacterial Viability Kit (Molecular Probe, Eugene, Oregon, USA). Stained membranes were mounted on glass slides (24 mm × 60 mm, thickness 0.17 m) with cover slips and analyzed utilizing confocal laser scanning microscopy (CLSM, LSM 810, Carl Zeiss, Germany). The COMSTAT program was employed to quantify CLSM images of biofilms formed on the membrane surface.

### 2.4 Microbial community analysis

For both the initial inoculum and samples obtained during Phase II of the three SBR reactors, microbial community analysis was undertaken. Total DNA extraction was carried out utilizing the Maxwell RSC PureFood GMO and Authentication Kit (Promega), following the manufacturer's protocol. PCR amplification was conducted using fusion primers targeting the V3 to V4 regions of the 16S rRNA gene with the extracted DNA. The quality and size of the PCR products were evaluated using a Bioanalyzer 2100 (Agilent, Palo Alto, CA, USA) equipped with a DNA 7500 chip. The resulting amplified DNA fragments were pooled, and sequencing was executed by CJ Bioscience, Inc. (Seoul, Korea), utilizing the Illumina MiSeq Sequencing system (Illumina, USA) in accordance with the manufacturer's guidelines. All these analytical processes were carried out on CJ Bioscience's bioinformatics cloud platform EzBioCloud (https://www.ezbiocloud.net/) employing 16S-based metagenome taxonomic profiling (MTP).

## 3 Results and discussion

### 3.1 Reactor performance under different OLR

**Fig. 2:**
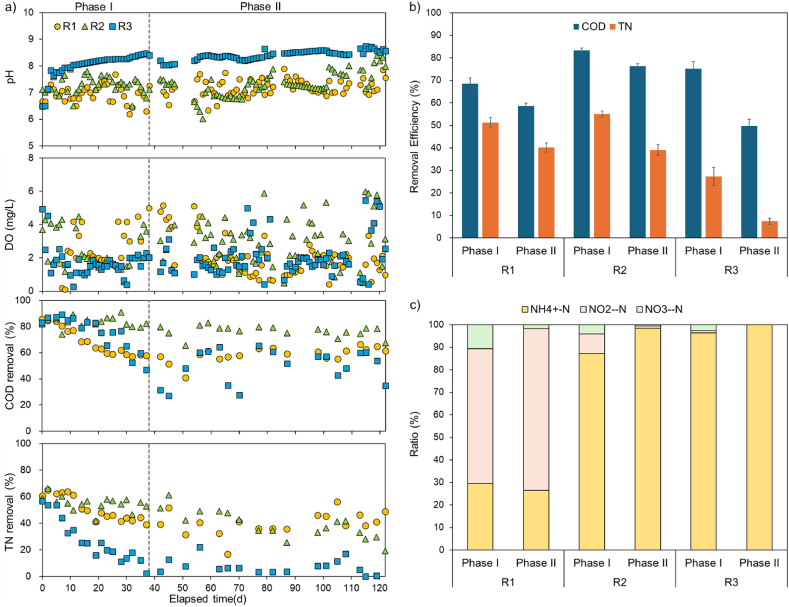
Changes in a) pH, DO, COD and TN removal during the whole process, b) average COD and TN removal at different operating conditions, c) NH_4_^+^-N, NO_2_^−^-N, NO_3_^−^-N ratio in effluent at different operating conditions.

Fig. 2a represents the monitoring of pH, DO, COD, and TN removal of R1, R2, and R3 throughout the entire operating period. DO was maintained within the range of 2–5 mg/L, and pH was also well controlled within the neutral range. However, R3 maintained a high pH due to insufficient nitrification. Fig. 2b illustrates the average COD and TN removal efficiency in R1, R2, and R3 across phases. Throughout the operation, the COD removal efficiency was highest in R2, ranging from 76.3 % to 83.4 % at an OLR of 0.33 kg COD/m^3^·day. Despite having the lowest OLR at 0.167 kg COD/m^3^·day, R1 exhibited lower removal efficiency ranging from 58.6 % to 68.5 %. In contrast to COD, TN removal efficiency ranged from 40.2 % to 51.2 % in R1 and was lowest in R3, ranging from 7.4 % to 27.4 %, even at the highest OLR. The low nitrogen removal in R3 could be attributed to the inhibition of nitrification by free ammonia at high pH [15].

In Phase II, where settling time was altered, both COD and TN removal efficiencies decreased across all conditions (Fig. 2b). This aligns with previous research findings where a reduction in settling time led to decreased COD and NH_4_^+^-N removal efficiencies [16]. This decrease in removal efficiency is attributed to the loss of sludge settling characteristics and PN content within EPS due to reduced settling time, coupled with the loss of heterotrophs and autotrophs. However, in the R1 condition with the lowest OLR, partial nitrification continued even after the phase change, indicating the presence of various microorganisms, including autotrophs, in the AGS structure, thus supporting the existing hypothesis of AGS formation (Fig. 2c). However, the proportion of nitrogen components in effluent changed with the phase change in the R1 condition. In Phase II conditions, the proportion of NO_2_^−^-N in the effluent of R1 ranged from 60 % to 72 %, indicating sufficient nitritation but continued inhibition of complete nitrification to NO_3_^−^-N. Further microbial analysis related to nitrogen removal will be discussed in section 3.4.

### 3.2 Effect of SBR operating control on sludge characteristics

**Fig. 3:**
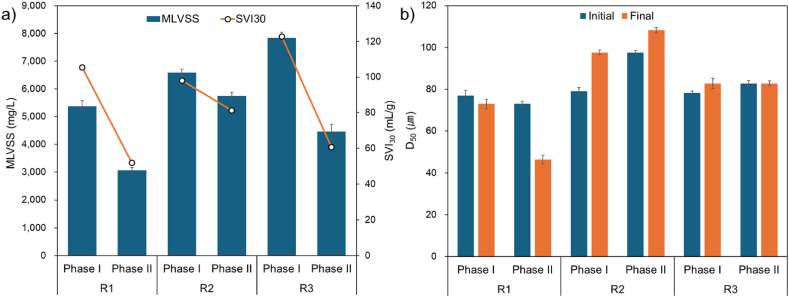
Changes in sludge characteristics at different operating conditions: a) MLVSS and SVI_30_, b) particle size.

Shortened settling times can enhance the development of favorable settling characteristics and dense AGS by eliminating sludge that hampers settling efficiency, primarily through generating higher hydraulic selective pressure [10]. In Fig. 3a, MLVSS and SVI_30_ were depicted to illustrate the relationship between biomass concentration and settling ability concerning variations in OLR and settling time across the SBR reactors. During Phase I, MLVSS increased with rising OLR. However, in Phase II, MLVSS decreased across all reactors as settling time diminished, a trend consistent with the observed changes in SVI_30_. The decline in MLVSS was attributed to the swift removal of sludge from the reactors prompted by abrupt alterations in operating conditions due to the shortened settling time. This outcome aligns with prior findings concerning biomass loss during the initial cultivation of AGS [17]. Ultimately, despite the microbial selection process due to the shortened settling time, this positively influenced sludge settling ability, as confirmed through SVI_30_ [18]. According to existing literature [19], the mature AGS's SVI_30_ ranges from 20 to 100 mL/g, varying greatly depending on experimental conditions, with an average of 30–50 mL/g. The results of this study were higher, indicating the formation of seed AGS rather than mature AGS. However, this was similar to the findings of Zou et al. [20], which reported a level of 71.1 ± 7.4 mL/g.

The development of AGS is directly influenced by changes in sludge particle size. Despite the decrease in MLVSS, R2 displayed an increase in average particle size (Fig. 3b). Additionally, while the average particle size of R3 remained relatively stable without a significant increase, considering the reduction in MLVSS, it suggests a gradual maturation of AGS. Conversely, in the case of R1, the average particle size decreased, likely due to the organic loading rate, particularly when compared to the greater reduction in MLVSS observed in R3. This observation aligns with previous findings suggesting that higher OLR speeds up AGS formation [3].

### 3.3 Effect of SBR operating control on EPS and sludge adhesion

**Fig. 4:**
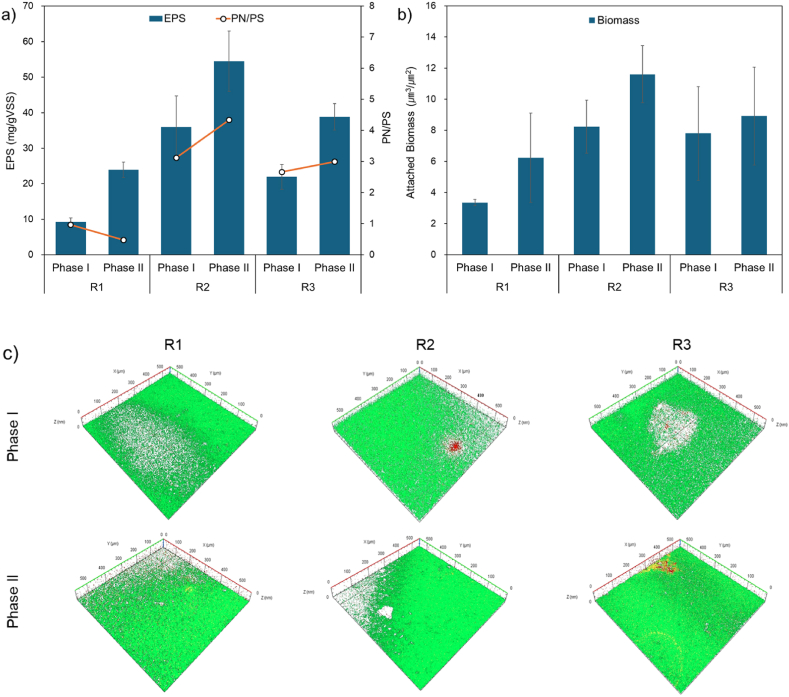
Changes in EPS and sludge adhesion at different operating conditions: a) EPS and PN/PS, b) attached biomass, and c) CLSM images of biofilm structure.

EPS resides within the extracellular matrix and microbial aggregates, serving as a pivotal element of microbial biofilms. It typically comprises organic compounds such as proteins (PN), polysaccharides (PS), humus, nucleic acids, lipids, and glycoproteins, exerting significant influence on material transport, floc formation, and stability [21]. In Fig. 4a, the fluctuations in EPS content and PN/PS composition are depicted throughout the operational period of the SBR. While EPS content decreased with higher OLRs, the PN/PS ratio was higher in R2 compared to R1 and R3. A higher PN/PS ratio generally fosters microbial cell accumulation and aggregation. PN can enhance cell attachment and floc formation by modifying cell surface hydrophobicity, whereas PS can form a backbone encapsulating bacteria, thus creating a network structure [22]. Previous studies have reported inconsistent effects of OLR on polysaccharide content in flocs, with some indicating no alteration and others showing a decrease in protein and an increase in polysaccharide with increasing OLR [23,24]. However, under low OLR conditions, the PN/PS ratio may not exhibit a linear relationship, and consequently, as EPS increases, SVI_30_ decreases. Therefore, higher EPS levels lead to improved sludge settling and accelerated AGS formation. Notably, the PN/PS ratio in R2, at 4.3, was higher than that in R1 and R3, suggesting that maintaining a PN/PS ratio around 4 ensures a favorable structure and aids in promoting microbial accumulation and aggregation, consistent with previous research findings [21].

Reducing settling time from Phase I to II resulted in increased EPS content in all reactors, aligning with previous reports indicating that shorter settling times promote the secretion of PN and PS compounds [10]. Particularly, in R2 and R3, the PN/PS ratio increased from 3.1 to 4.3 and from 2.7 to 3.0, respectively, indicating progressive AGS maturation. However, in R1, with the lowest OLR, while EPS content increased, there was no significant change in the PN/PS ratio. This contrasts with previous findings that suggest under prolonged low OLR conditions, sludge microbes may be in a starved state, leading to the breakdown of EPS matrices for exogenous metabolism, with PS content increasing more than PN [25]. Unlike PN, PS, despite being hydrophilic, can promote the formation of a stable floc structure by distributing throughout the floc and building a mesh skeleton [26]. However, PS is less supportive of AGS structure stability compared to PN [21], indicating a distinct difference in microbial attachment properties.

CLSM analysis is a method for assessing whether the generated EPS can bind or aggregate through contact with each other on hydrophilic membranes under different OLR and settling time conditions. The volume of actual attached biomass per membrane area increased with shortened settling time from Phase I to II under all experimental conditions (Fig. 4b). Microbial attachment was particularly proportional to EPS content and PN/PS ratio rather than OLR. This illustrates that as settling time decreases, microbes secrete abundant hydrophobic PN, thereby enhancing microbial cell aggregation and settling efficiency. In Fig. 4c, the results of image analysis at the center of the membrane show that all attached biomass under all experimental conditions exhibited strong green fluorescence indicative of high metabolic activity in live cells. Particularly, R2, with the highest attachment, exhibited denser green fluorescence compared to other experimental conditions, consistent with superior attachment characteristics.

**Fig. 5:**
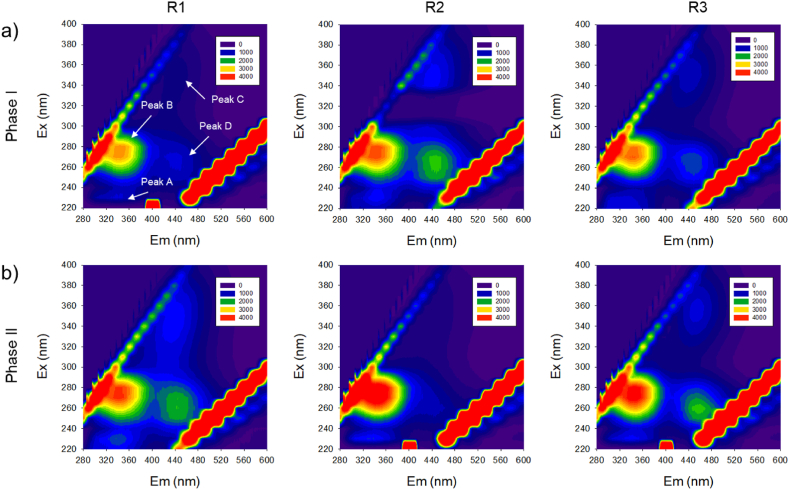
EEM fluorescence spectra of EPS at different operating conditions: a) Phase I, b) Phase II.

Changes in the organic composition of EPS were analyzed using F-EEM fluorescence spectroscopy (Fig. 5). The F-EEM spectrum provides specific information about tryptophan protein-like (Peak A, Ex/Em = 220–240/330-360), aromatic protein-like (Peak B, Ex/Em = 270–280/330-360), humic acid-like (Peak C, Ex/Em = 300–340/400-450), and fulvic acid-like (Peak D, Ex/Em = 230–260/400-450) substances within sludge EPS. Throughout the entire SBR operation, significant changes in tryptophan protein-like (Peak A) and aromatic protein-like (Peak B) substances were observed in all sludge samples (R1, R2, R3), indicating their prominence as major components of EPS. Guo et al. [27] noted that aromatic and tryptophan protein-like substances contribute to sludge structure formation through surface charge regulation.

During Phase II, where settling time was reduced, there was an increase in the intensity of Peak B observed under all conditions. This corresponds with the earlier finding of increased PN ratio within EPS and suggests that shorter settling time promotes microbial aggregation, crucial for granule formation [28]. Unlike R1 and R2, humic acid and fulvic acid-like substances were not observed in Phase II R2, which had the highest PN/PS ratio. Humic acid-like substances are associated with the growth of specific bacteria, degradation of macromolecular organic matter (e.g., PS and PN), and decomposition of dead cells [29]. The decrease in humic acid in R2 may be attributed to the consumption of PS during granulation, indicating maturation of the granular structure [30]. In the initial stages of granulation, PN is predominantly distributed on the surface of AGS, but after maturation, PS is more widely distributed, playing a crucial role in maintaining the granular structure [31]. Thus, these differences demonstrate that while R1 and R3 have entered a mature stage, R2 is in the stage of progressing towards maturity, indicating that even under low OLR conditions, AGS can operate stably.

### 3.4 Characteristics of microbial community analysis

#### 3.4.1 Alpha diversity analysis

To explore the microbial characteristics of aerobic granular sludge and various SBRs in Phase II, high-throughput sequencing analysis of 16S rDNA amplicon technology was conducted. A total of 974–1326 OTUs were identified with over 99.4 % Good's coverage, indicating accurate interpretation of species abundance and diversity from the sequencing results. Alpha diversity metrics, including ACE and Chao1 for microbial community richness, and Shannon and Simpson for microbial community evenness, were employed to assess microbial clustering differences, as summarized in Table 2. In Phase II, all samples exhibited a lower Shannon index and a higher Simpson index compared to the initial phase, suggesting a potential decrease in microbial community diversity within the sludge. Particularly notable numerical changes were observed in R2 at an OLR of 0.33 kg COD/m^3^·day, indicating a significant impact on microbial clustering. This finding is consistent with similar observations by Liu et al. [32], suggesting that the granulation process may diminish microbial community diversity within the sludge. In contrast, ACE and Chao1 indices were lower only in R2 compared to aerobic granular sludge, implying a decrease in the uniformity of microbial clustering within the sludge during the granulation process.

**Table 2:** Alpha diversity indices characterizing sludge samples.

Sludge samples	OTUs	ACE	Chao1	Shannon	Simpson	Good's coverage
Inoculum	1069	1219.23	1156.02	4.53	0.04	0.9936
R1	1326	1399.62	1349.26	3.88	0.06	0.9979
R2	974	1109.04	1043.20	3.28	0.12	0.9966
R3	1125	1237.01	1174.08	3.83	0.06	0.9972

#### 3.4.2 Microbial community structure analysis

**Fig. 6:**
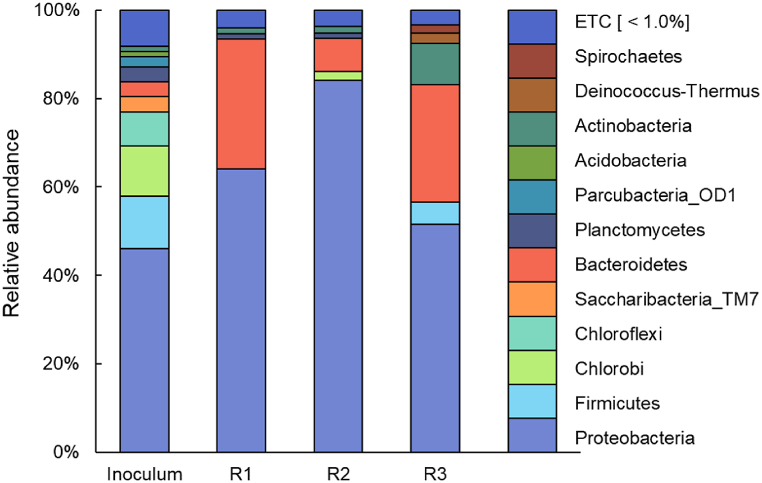
Relative abundance of phyla bacterial classes.

To investigate shifts in microbial community composition in response to OLR variations, microbial community analysis was conducted during Phase II. The graph illustrates the microbial community composition of reactors at the phylum level based on OLR (Fig. 6). Overall, *Proteobacteria* were the dominant phylum, constituting 46.1 % in the inoculum and 64.1 %, 84.1 %, and 51.6 % in R1, R2, and R3, respectively, during Phase II. The *Proteobacteria* phylum plays a crucial role in organic and nitrogen removal and may contribute to the efficiency of AGS pollutant removal. Additionally, it is known to secrete abundant EPS, which can promote granulation by providing important metabolic diversity [33]. This finding is consistent with earlier results on organic removal efficiency, as reactor R2, which had the highest proportion of *Proteobacteria*, exhibited the highest COD removal efficiency, while reactor R3, with the lowest proportion of *Proteobacteria*, showed the lowest organic removal efficiency. The phylum *Bacteroidetes* is primarily involved in processes such as denitrification, accounting for only 3.4 % in the inoculum. However, following OLR variations in Phase II, it increased to 29.3 %, 7.5 %, and 26.7 % in R1, R2, and R3, respectively, indicating that OLR changes promoted the growth of the *Bacteroidetes* phylum.

In the microbial community analysis at the genus level, as shown in Table 3, the *Nitrosomonas* genus, known as ammonia oxidation bacteria (AOB), was detected at 7.6 % and 1.8 % in reactors R1 and R2, respectively. This supports previous findings regarding partial nitrification. Conversely, *Nitrosomonas* was not detected in R3, correlating with the lowest ammonia nitrogen removal among the three conditions. Nitrite oxidation bacteria (NOB) were not detected in any of the reactors, suggesting an operation different from the typical nitrite oxidation-nitrate reduction pathway. The *Comamonas* genus, along with the *Paracoccus* genus, was dominant in the final stabilized AGS [34,35], with percentages of 3.9 % and 45.1 % in R1 and R2, respectively. The *Thauera* genus, representing denitrifying bacteria, showed relatively consistent abundance across the inoculum, R1, R2, and R3, ranging from 13.9 % to 19.0 % after OLR variation. The *Pseudomonas* genus, known for its involvement in various processes such as denitrification, showed the highest percentage in Phase II's R3 (10.4 %) and was detected at 2.5 % in R2. *Aequorivita*, *Corynebacterium*, *Thiopseudomonas*, and *Zoogloeaceae* are known as denitrifying bacteria (DNB), accounting for 3.6 %, 5.4 %, 1.7 %, and 1.2 %, respectively, in R3, with *Zoogloeaceae* recognized for its role in cell aggregation and EPS production [36]. Glycogen-accumulating organisms (GAO), represented by *Caldimonas* in the inoculum at 5.5 %, decreased to 1.6 % in R, with *Alkalispirochaeta*, *Prolixibacteraceae*, and *Sphingobacteriaceae* appearing. GAO bacteria were most detected in R1, with *Saprospiraceae* and *Chitinophagaceae* comprising 5.4 % and 3.9 %, respectively.

**Table 3:** Heatmap presenting the evolution in dominant bacterial genera.

Nutrients removal microbe at genus level		Relative abundance (%)			
		Inoculum	R1	R2	R3
AOB	*Nitrosomonas*	0.0	7.6	1.8	0.0
DNB	*Azoarcus*	5.0	0.0	0.0	0.0
	*Comamonas*	0.0	3.9	45.1	0.0
	*Ottowia*	0.0	12.2	5.0	0.0
	*Paracoccus*	0.0	0.0	0.0	3.4
	*Thauera*	16.7	19.0	13.9	15.7
	*Zoogloeaceae_uc*	0.0	0.0	0.0	1.2
	*Aequorivita*	0.0	0.0	0.0	3.6
	*Castellaniella*	0.0	1.3	0.0	0.0
	*Corynebacterium*	0.0	0.0	0.0	5.4
	*Thiopseudomonas*	0.0	0.0	0.0	1.7
DPAO	*Pseudomonas*	0.0	0.0	2.5	10.4
GAO	*Alkalispirochaeta*	0.0	0.0	0.0	1.8
	*Caldimonas*	5.5	0.0	0.0	1.6
	*Chitinophagaceae_uc*	0.0	3.9	0.0	0.0
	*Prolixibacteraceae_uc*	0.0	0.0	0.0	1.5
	*Saprospiraceae_uc*	0.0	5.4	1.3	0.0
	*Sphingobacteriaceae_uc*	0.0	0.0	0.0	2.6

## 4 Conclusions

The optimization of short settling time in SBR operational parameters was found to enhance settling efficiency and promote dense AGS formation. As settling time decreased, the sludge exhibited improved settling ability, facilitating the selection of favorable microorganisms during the granulation process. Furthermore, the variation in EPS content was confirmed to be pivotal in sludge aggregation and AGS formation. Specifically, the increase in the PN/PS ratio within EPS positively influenced sludge attachment, contributing to the progressive maturation of AGS. Performance assessment of reactors under varying OLR conditions revealed that while COD removal efficiency was high at an OLR of 1 kg COD/m^3^·day, nitrogen removal efficiency was low. This was attributed to changes in microbial community composition, particularly fluctuations in the proportions of *Proteobacteria* and *Bacteriodetes* phyla, which elucidated the reasons for the differences in removal efficiency. Additionally, analysis of microbial diversity and community composition highlighted the impact of OLR variations on the diversity and structure of microbial communities. These findings underscore the significant influence of SBR operational strategies on the performance and biological stability of AGS processes, offering valuable insights for the efficient operation of high-strength wastewater treatment processes in the future.

## CRediT authorship contribution statement

**Kyung Jin Min:** Conceptualization, Supervision, Writing – original draft. **Eunyoung Lee:** Investigation, Visualization, Writing – original draft. **Ah Hyun Lee:** Formal analysis, Methodology. **Do Yeon Kim:** Formal analysis, Methodology. **Ki Young Park:** Supervision, Writing – review & editing.

## Declaration of competing interest

The authors declare that they have no known competing financial interests or personal relationships that could have appeared to influence the work reported in this paper.
